# Ionized magnesium levels and atrial fibrillation in patients undergoing cardiac surgery – The iMagic Prospective Cohort Study

**DOI:** 10.1371/journal.pone.0345860

**Published:** 2026-04-02

**Authors:** Mahan Sadjadi, Thilo von Groote, Sina Krone, Ludwig Maximilian Schöne, Christian Strauß, Moritz J. Mertes, Christine Martin, Felix Dinkel, Jessica Schellnack, Thomas Fraune, Carola Wempe, Alexander Zarbock, Hendrik Booke, Antje Gottschalk

**Affiliations:** 1 Department of Anesthesiology, Intensive Care and Pain Medicine, University Hospital Münster, Münster, Germany; 2 Life Systems Medizintechnik-Service GmbH, Mönchengladbach, Germany; 3 Department of Anesthesiology, Intensive Care and Pain Medicine, Florence-Nightingale-Hospital, Düsseldorf, Germany; Scuola Superiore Sant'Anna, ITALY

## Abstract

**Background:**

New-onset atrial fibrillation (AF) after cardiac surgery is associated with unfavorable outcomes. Electrolyte imbalances have been described as potential risk factors. The effects of ionized magnesium (iMg), a component of total magnesium (tMg) concentration in the blood, and the influence of iMg-dynamics on the incidence of postoperative atrial fibrillation (POAF) are unclear.

**Methods:**

Ionized Magnesium Levels and Atrial Fibrillation in Patients undergoing Cardiac surgery (iMagic) was a single-center prospective cohort study. A total of 200 patients were enrolled over six months. Plasma-iMg and -tMg levels were measured at different time points in patients without pre-existing AF undergoing on-pump cardiac surgery. The primary outcome was the incidence of POAF.

**Results:**

Of 200 participants, 55 (27.5%) developed new-onset POAF. Ionized magnesium levels at five pre-specified time points did not vary significantly between patients who later developed POAF and those who did not. Exploratory analyses showed a trend toward higher postoperative iMg, but not tMg (0.78 mmol/L, standard deviation [SD] 0.1 vs. 0.74 mmol/L, SD 0.11, P = 0.096) in patients who developed POAF. This exploratory finding was more pronounced in patients who underwent isolated coronary artery bypass graft (CABG) surgery, and at certain time points, including anesthesia induction (mean iMg 0.71 mmol/L, SD 0.11 vs. 0.65 mmol/L, SD 0.07, P = 0.05).

**Conclusions:**

Plasma-iMg levels did not vary between cardiac surgery patients who developed POAF and those who did not. Exploratory subgroup analyses indicated that iMg-levels may be associated with POAF in patients undergoing isolated CABG surgery. Perioperative measurement of iMg in patients at risk of POAF undergoing isolated CABG surgery may be beneficial, but more data are needed to support our preliminary findings. The risk factors for dysmagnesemia and subsequent patient-centered outcomes, such as new-onset POAF, have yet to be described for cardiac surgery.

## Introduction

Patients undergoing cardiac surgery are at a high risk of perioperative complications, including postoperative atrial fibrillation (POAF), one of the most common adverse events, with incidence rates ranging from 10 to 50%. [1–4] Since POAF can lead to significant morbidity and mortality [3–6], prevention is essential to improve outcomes. [7] The mechanistic basis of POAF remains an active area of research, with inflammatory processes, oxidative stress, changes in the autonomic nervous system, and changes of the membrane potential of cardiomyocytes recognized as important influencing factors. [8] One group of factors proposed as potential causes of POAF are electrolyte imbalances, such as hypomagnesemia, which might influence membrane potential. [9] The effect of perioperative magnesium administration has been studied in several randomized trials over the past few years. [10–12] However, such studies focused on total Magnesium levels (tMg) and did not investigate the levels of ionized magnesium (iMg), the physiologically active form. Thus, any effect these levels may have on the incidence of new-onset arrhythmias remains unclear. [13] In this study we investigate the association between serum magnesium levels at different time points and the occurrence of POAF following cardiac surgery, aiming to better describe the role of magnesium in the incidence of POAF, and to identify suitable time points for measurement and intervention.

## Methods

### Design and ethics

The Ionized Magnesium Levels and Atrial Fibrillation in Patients undergoing Cardiac surgery (iMagic) Study was a single-center prospective cohort study. Ethical approval was obtained from the Research Ethics Committee of the Chamber of Physicians, Westfalen-Lippe, and the University of Münster (2022–277-f-S). The study was registered before its initiation in the German Clinical Trials Register (DRKS00029391), and was conducted in accordance with the Declaration of Helsinki guidelines. Reporting adheres to the STROBE guidelines.

### Patient recruitment and consent

Following verbal and written informed consent, eligible adult patients (age ≥ 18 years) without atrial fibrillation and scheduled to undergo open-chest cardiac surgery with cardiopulmonary bypass were included in the study. Patients were excluded if they were dependent on the investigator, or if they were held in an institution by legal or official orders. No patients were excluded from the study based on sex, ethnicity, religion or other individual factors. Recruitment commenced on 24 August 2022 and was completed on 9 February 2023.

### Study procedures

Blood samples were collected from all patients for iMg measurements at five different time points, as follows:

1. Upon induction of general anesthesia
2. Before the start of extracorporeal circulation
3. Before termination of cardiopulmonary bypass (CPB)
4. At intensive care unit (ICU) admission
5. 24 hours after surgery

Measurement of iMg was performed with a Stat Profile Prime® Plus blood gas analyzer (Nova Biomedical Corporation, USA) using waste blood drawn for standard arterial blood gas analysis. The given reference values for tMg and iMg were 0.6–1.1 mmol/L and 0.45–0.59 mmol/L respectively. All patients received 20 ml of magnesium chloride 10% with release of the aortic clamp. In our cohort, three types of cardioplegia solutions were used: Custodiol HKT® (electrolyte solution), Calafiore’s cardioplegia (blood based), and Buckberg’s cardioplegia (blood based). The observation period was up to 7 days after major surgery or until ICU-discharge, whatever occurred first. All patients received continuous monitoring according to local ICU standards. This included continuous ECG monitoring in all patients over the entire observational period. Treatment of all patients was done according to local standard operating procedures.

### Data collection

Data were collected as electronic files and were confidentially stored in de-identified forms on secure servers of the University of Münster (Germany).

### Outcomes

The primary outcome was the incidence of POAF, detected by using electrocardiography within seven days of surgery, or upon ICU discharge, whichever occurred first. All episodes of atrial fibrillation post-surgery, regardless of their duration, were defined as POAF. Analyses were conducted for the entire cohort and the a priori subgroup of patients who underwent isolated coronary artery bypass graft (CABG) surgery.

### Sample size

Given that the incidence rates of POAF after cardiac surgery range from 10 to 50%, with an estimated average of 30%, a sample size calculation was performed with the goal of detecting a clinically meaningful difference in POAF incidence (≥ 10% between groups with different iMg levels). The required sample size, with a significance level of α = 0.05 and a power of 1 – β = 0.80, was estimated to be 142 patients. A sample size of 200 patients increased statistical power for exploratory analyses and provided an estimated margin of error of approximately 6.35% when calculating the POAF incidence, ensuring a reasonable degree of precision for the primary outcome.

### Statistical analysis

Descriptive statistics for variables of interest were summarized using mean and standard deviation (SD), or median and quartiles, based on variable characteristics. Binary variables were recorded as frequencies and percentages. Univariate logistic regression was used to analyze the association between measured iMg values and new-onset POAF. Multivariate analyses were foregone due to the exploratory nature of our analyses and resulting statistical limitations.

## Results

**Fig 1:**
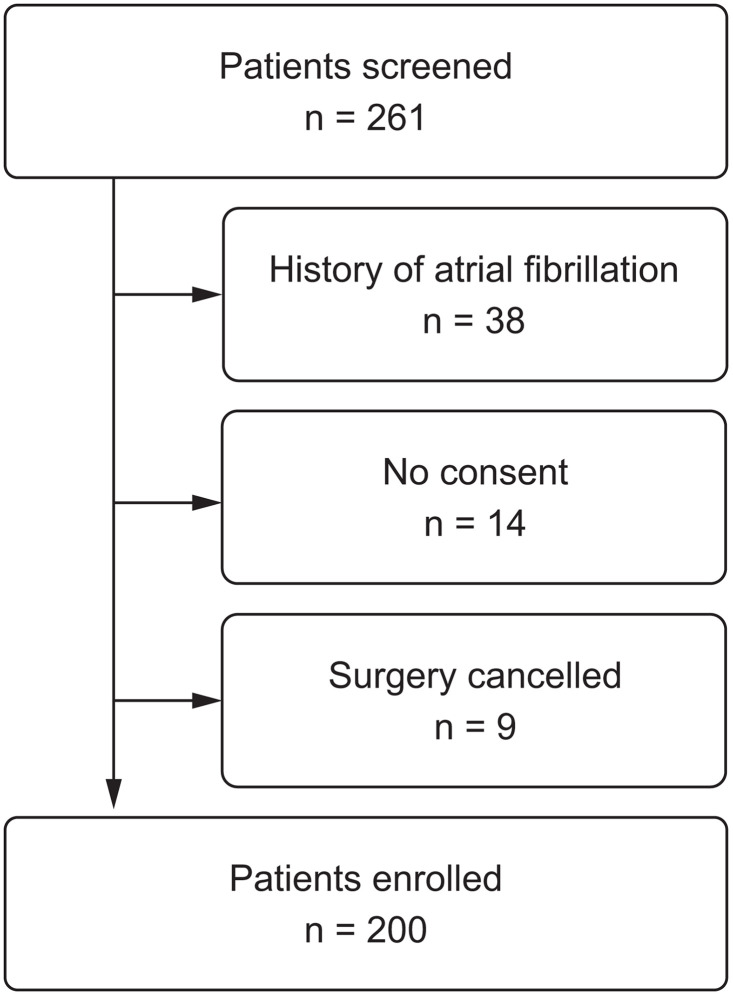
Participant flowchart.

Two-hundred patients were enrolled in our study between August 2022 and February 2023 (Fig 1). The mean age of the participants was 67 years (SD 10.7 years), with 143 (70.8%) males. Approximately half of the patients underwent valve repair or replacement surgeries (57%), whereas the remaining patients underwent elective CABG. The baseline characteristics of the patients are summarized in Table 1.

**Table 1:** Demographic data.

Demographic data	Total (n = 200)	Postoperative atrial fibrillation (n = 55)	No postoperative atrial fibrillation (n = 145)
Age, mean (SD), years	67 (10.7)	70 (7.9)	66 (11.4)
Male sex, no. (%)	143 (70.8)	37 (67.3)	105 (72.9)
BMI, mean (SD)	27.7 (5.9)	27.6 (6.8)	27.8 (5.5)
Euroscore, median (Q1-Q3)	2.4 (1.3-5.4)	4.6 (2.4-9.8)	2.0 (1.3-4.5)
**Comorbidities, no. (%)**			
Smoking	49 (24.5)	13 (23.6)	36 (24.8)
Hypertension	155 (77.5)	44 (80)	111 (76.6)
Diabetes	55 (27.5)	17 (31)	38 (26.2)
Chronic heart failure NYHA I NYHA II NYHA III NYHA IV	67 (33.5) 12 (6) 21 (10.5) 32 (16) 2 (1)	27 (49.1) 6 (10.9) 8 (14.5) 13 (23.6) 0	40 (27.6) 6 (10.9) 13 (6.5) 19 (9.5) 2 (1)
Coronary artery disease	99 (49.5)	32 (58)	67 (46.2)
Peripheral artery disease	21 (10.5)	8 (14.5)	13 (6.5)
Chronic obstructive pulmonary disease	15 (7.5)	5 (9.1)	10 (6.9)
Chronic kidney disease (eGFR < 60 ml/min/1.73m²)	26 (13)	10 (18.2)	16 (11)
**Medications, no. (%)**			
Statins	106 (53)	30 (54.5)	76 (52.4)
Nonsteroidal anti-inflammatory drugs	72 (36)	12 (21.8)	60 (41.4)
Beta blockers	106 (53)	33 (60)	73 (50.3)
ACEi / ARB	138 (69)	42 (76)	96 (66.2)
Calcium antagonists	72 (36)	15 (27.3)	57 (39.3)
Diuretics	76 (38)	27 (49)	49 (33.8)
**Surgical data**			
Surgery including repair or replacement of valves, no. (%)	114 (57.0)	36 (65.0)	78 (53.8)
Duration of surgery, median (Q1-Q3), min	230.5 (195.0-280.5)	241.5 (216.5-314.0)	222 (189.0-276.0)
Duration of aortic cross-clamp, median (Q1-Q3), min	84 (63.0-119.0)	93.5 (70.6-127.0)	82 (60.0-113.0)
Duration of CPB, median (Q1-Q3), min	125.5 (100.0-166.5)	142.5 (108-170.3)	121 (96.0-162.5)
Number of defibrillations at end of CPB, median	1	1	1

Abbreviations: ACEi, Angiotensin-Converting-Enzyme Inhibitors; ARB, Angiotensin-Receptor Blockers; CPB, Cardiopulmonary Bypass; eGFR: estimated glomerular filtration rate; NYHA, New York Heart Association; SD, Standard Deviation.

Overall, 55 patients (27.5%) developed POAF. Measurements of magnesium levels at the five pre-specified time points in all patients showed no statistically significant differences between patients who ultimately developed POAF and those who did not. However, there was a notable difference in the pre-specified subgroup of patients undergoing isolated CABG-surgery (baseline characteristics: S1 Table), where patients who developed new-onset POAF had higher mean plasma iMg concentrations at anesthesia induction, compared to those who did not develop POAF (mean iMg 0.71 mmol/L (SD 0.11) vs. 0.65 mmol/L (SD 0.07), P = 0.05). Concentrations of tMg at the same time point did not differ notably (mean tMg 0.86 mmol/L (SD 0.12) vs. 0.83 mmol/L (SD 0.07), P = 0.38). The outcomes of the whole cohort and subgroup analyses are summarized in Table 2.

**Table 2:** Primary outcome.

	Post-OP Atrial Fibrillation (n = 55)	No Post-OP Atrial Fibrillation (n = 145)	p-value
**All patients**			
Plasma tMg at anesthesia induction, mean (SD), mmol/L	0.90 (0.46)	0.84 (0.07)	0.34
Plasma iMg at anesthesia induction, mean (SD), mmol/L	0.67 (0.09)	0.68 (0.10)	0.23
Plasma iMg before regular administration of magnesium chloride at start of CPB, mean (SD), mmol/L	0.78 (0.19)	0.82 (0.24)	0.30
Plasma iMg before termination of CPB, mean (SD), mmol/L	1.12 (0.21)	1.12 (0.19)	1
Plasma iMg at ICU admission, mean (SD), mmol/L	0.89 (0.16)	0.92 (0.20)	0.57
Plasma iMg 24 hours after surgery, mean (SD), mmol/L	0.74 (0.11)	0.78 (0.10)	0.10
Plasma tMg 24 hours after surgery, mean (SD), mmol/L	0.93 (0.13)	0.96 (0.12)	0.34
**Patients undergoing isolated coronary artery bypass graft surgery**			
Plasma tMg at anesthesia induction, mean (SD), mmol/L	0.83 (0.07)	0.86 (0.12)	0.38
Plasma iMg at anesthesia induction, mean (SD), mmol/L	0.65 (0.07)	0.71 (0.11)	0.05*
Plasma iMg before regular administration of magnesium chloride at start of CPB, mean (SD), mmol/L	0.76 (0.20)	0.79 (0.27)	0.60
Plasma iMg before termination of CPB, mean (SD), mmol/L	1.05 (0.20)	1.06 (0.24)	0.92
Plasma iMg at ICU admission, mean (SD), mmol/L	0.84 (0.14)	0.79 (0.22)	0.64
Plasma iMg 24 hours after surgery, mean (SD), mmol/L	0.71 (0.08)	0.76 (0.09)	0.19
Plasma tMg 24 hours after surgery, mean (SD), mmol/L	0.90 (0.12)	0.92 (0.12)	0.62

Abbreviations: CPB, Cardiopulmonary Bypass; iMg, Ionized Magnesium; tMg, Total Magnesium; SD, Standard Deviation. Reference values for tMg: 0.6–1.1 mmol/L and iMg: 0.45 – 0.59 mmol/L.

## Discussion

No significant differences were observed in our study regarding iMg levels at any of the pre-specified time points or in the dynamics of magnesium levels between patients who developed POAF and those who did not, across a heterogeneous cohort of 200 patients undergoing open-chest cardiac surgery with cardiopulmonary bypass. We observed large interpersonal variations in plasma iMg levels throughout treatment course in our cohort. In patients who developed POAF, a trend toward higher mean postoperative iMg, but not tMg, levels was observed.

Exploratory subgroup analyses revealed that iMg levels could be indicative of an elevated risk for development of POAF in patients undergoing isolated CABG surgeries. In these patients, magnesium, as well as imbalances in magnesium levels can play a crucial role in the development of POAF [14,15]. In patients undergoing surgeries involving the electrophysiological structures of the heart, such as valve replacement, magnesium does not appear to play a key role. This could, in part, be due to the fact that structural changes affect the cardiac conductive system of the heart and may be the primary driver of POAF in this cohort, indicating a pathophysiologically different phenotype of POAF. The association between valve surgeries and disruption to the heart's conduction system has been well established [16], as has the fact that this association represents an individual risk factor for the development of POAF. These patients developed arrhythmias due to causes other than electrolyte imbalance. In our cohort, more than half of the patients underwent valve replacement or combined surgeries, which may hence have masked any notable effects across the entire cohort. Our data indicate that further investigation of the potential association between abnormal iMg levels and the onset of POAF is warranted, especially in patients undergoing CABG surgery. In these patients, there is a potential that measuring iMg levels offers added benefits over measuring tMg alone. However, the relatively small size of our cohort and the lack of statistical power to conclusively demonstrate this, precludes any definitive statement, and further research is needed. The role of serum magnesium levels has been discussed in the context of atrial fibrillation and other arrhythmias, both after cardiac surgery and in nonsurgical patients, mostly with regard to the administration of magnesium as a therapeutic agent. [9,17–20] In most studies to date, the target variable has been tMg concentration, and the findings are that hypomagnesemia is associated with new-onset POAF. Our data suggest that the pathophysiology might be more complicated, and more research is needed to validate the role of magnesium and its administration in enhancing perioperative safety for patients at risk of developing new-onset POAF. In addition, the risk factors for imbalance in iMg levels in patients undergoing cardiac surgery are not well defined, and require further investigation. Given that iMg is the electrophysiologically active form and can be measured at the point-of-care, a case could be made for it being at the center of physicians’ and researchers’ considerations regarding magnesium status and supplementation in patients at risk of POAF. [21,22]

Our study has several limitations that have to be considered when interpreting the results. First, this was a small exploratory study without prespecified power calculation and results should strictly be considered as hypothesis-generating. Further, patients with POAF and those without differed on some baseline variables (e.g., for baseline Euroscore and CKD-incidence). Also, no long-term follow-up of patients was done and we do not provide data on POAF incidence at hospital discharge. However, this study also has its strengths as iMg-levels and POAF in cardiac surgery patients have not yet been studied conclusively. Hence, this study provides preliminary data on iMg levels that can be anticipated at different time points in patients undergoing cardiac surgery, as well as data on dynamic changes, offering insight into time windows for potential interventions. These findings can be used in future studies to help demonstrate the effects of iMg levels on the occurrence of POAF and further patient-centered outcomes, depending on individual risk profiles. Therefore, future trials should adopt individualized intervention designs, aligned with the tenets of precision medicine to determine when, in whom, and how much magnesium should be administered and which iMg levels to target.

## Supporting information

S1 Table Baseline characteristics of the subgroup of patients undergoing isolated CABG surgery. (DOCX)
