# Supplementary material for: Ionized magnesium levels and atrial fibrillation in patients undergoing cardiac surgery – The iMagic Prospective Cohort Study
Source: PLoS One. 2026 Apr 2;21(4):e0345860. doi: 10.1371/journal.pone.0345860 (PMC13046244; doi:10.1371/journal.pone.0345860)
Supplement: S1 Table — (DOCX) [file pone.0345860.s001.docx]

**Supplementary table 1. Baseline characteristics of the subgroup of patients undergoing isolated CABG surgery**

| **Demographic data** | **Total, (n=86)** | **Post-OP Atrial Fibrillation (n=19)** | **No Post-OP Atrial Fibrillation (n=67)** |
| --- | --- | --- | --- |
| Age, mean (SD), years | 68 (10) | 70 (7) | 67 (10) |
| Male sex, no. (%) | 69 (80) | 15 (79) | 54 (81) |
| BMI, mean (SD) | 28.2 (5.8) | 26.7 (6.3) | 28.6 (5.6) |
| Euroscore, median (Q1-Q3) | 1.8 (1.2, 4.3) | 3.1 (2.1, 5.5) | 1.5 (1.1, 3.4) |
| **Comorbidities, no. (%)** | | | |
| Smoker | 32 (37) | 8 (42) | 24 (36) |
| Hypertension | 75 (87) | 15 (79) | 60 (90) |
| Diabetes mellitus | 30 (35) | 6 (32) | 24 (36) |
| Chronic Heart Failure  NYHA I  NYHA II  NYHA III  NYHA IV | 6 (7)  4 (5)  16 (19)  1 (1) | 1 (5)  4 (21)  5 (26)  0 (0) | 5 (8)  0 (0)  11 (16)  1 (2) |
| Coronary Artery Disease | 86 (100) | 19 (100) | 67 (100) |
| Peripheral Artery Disease | 20 (23) | 6 (32) | 14 (21) |
| COPD | 10 (12) | 3 (16) | 7 (11) |
| Chronic Kidney Disease (eGFR < 60 ml/min/1.72m^2^) | 16 (19) | 6 (32) | 10 (15) |
| **Medications, no. (%)** | | | |
| Statins | 62 (72) | 14 (74) | 48 (72) |
| NSAIDs | 48 (56) | 5 (26) | 43 (64) |
| Beta blockers | 51 (59) | 11 (58) | 40 (60) |
| ACEi/ARB | 66 (77) | 15 (79) | 51 (76) |
| Calcium antagonists | 35 (41) | 5 (26) | 30 (45) |
| Diuretics | 36 (42) | 11 (58) | 25 (37) |
| **Surgical Data** | | | |
| Duration of Surgery, median (Q1-Q3), min | 220 (190, 260) | 237 (207, 252) | 219 (190, 258) |
| Duration of Aortic Cross-Clamp, median (Q1-Q3), min | 65 (51, 84) | 70 (49, 83) | 63 (52, 84) |
| Duration of Cardio-pulmonary Bypass, median (Q1-Q3), min | 105 (92, 128) | 108 (98, 141) | 102 (87, 127) |
